# DNA-nanoparticle assemblies go organic: Macroscopic polymeric materials with nanosized features

**DOI:** 10.1186/1477-3155-10-21

**Published:** 2012-05-30

**Authors:** Elad D Mentovich, Konstantin Livanov, Deepak K Prusty, Mukules Sowwan, Shachar Richter

**Affiliations:** 1 Faculty of Exact Sciences and Center for Nanoscience and Nanotechnology, Tel-Aviv University, Ramat Aviv, Tel-Aviv, 69978, Israel; 2 University of Groningen, Zernike Institute for Advanced Materials, Nijenborgh 4, 9747, AG, Groningen, The Netherlands; 3 Nanotechnology Research Laboratory, Materials Engineering Department, Al-Quds University, East Jerusalem, Palestinian Authority

**Keywords:** DNA block copolymer, DNA-Nanoparticle Assemblies, Self- Organization, Micelles

## Abstract

**Background:**

One of the goals in the field of structural DNA nanotechnology is the use of DNA to build up 2- and 3-D nanostructures. The research in this field is motivated by the remarkable structural features of DNA as well as by its unique and reversible recognition properties. Nucleic acids can be used alone as the skeleton of a broad range of periodic nanopatterns and nanoobjects and in addition, DNA can serve as a linker or template to form DNA-hybrid structures with other materials. This approach can be used for the development of new detection strategies as well as nanoelectronic structures and devices.

**Method:**

Here we present a new method for the generation of unprecedented ***all-organic*** conjugated-polymer nanoparticle networks guided by DNA, based on a hierarchical self-assembly process. First, microphase separation of amphiphilic block copolymers induced the formation of spherical nanoobjects. As a second ordering concept, DNA base pairing has been employed for the controlled spatial definition of the conjugated-polymer particles within the bulk material. These networks offer the flexibility and the diversity of soft polymeric materials. Thus, simple chemical methodologies could be applied in order to tune the network's electrical, optical and mechanical properties.

**Results and conclusions:**

One- two- and three-dimensional networks have been successfully formed. Common to all morphologies is the integrity of the micelles consisting of DNA block copolymer (DBC), which creates an all-organic engineered network.

## Background

The use of DNA to build 2- and 3-D nanostructures is based on its remarkable structural features as well as on its unique and reversible recognition properties. In this newly-established field, called structural DNA nanotechnology, nucleic acids can be used alone as the skeleton of a broad range of periodic nanopatterns and nanoobjects [1-5]. In addition, DNA can serve as a linker or template to form DNA-hybrid structures with other materials [6-9]. DNA utilized in this context leads to new detection strategies [10,11] as well as nanoelectronic structures [12] and devices [13].

While many examples in the field of structural DNA nanotechnology, like the ones presented above, deal with discrete objects or 2-D periodic structures not exceeding the size of one micron, much less attention has been devoted to bulk-DNA materials of macroscopic dimensions exhibiting nano-structured features. Again, one can distinguish between structures composed exclusively of nucleic acids like DNA hydrogels, and networks consisting of both synthetic polymers and nucleic acids. The building blocks of pristine DNA networks are branched, double-stranded (ds) DNA crossovers, which are covalently connected by DNA ligase. They are biocompatible, biodegradable, inexpensive to fabricate and easily molded into desired shapes and sizes. The salient features of such hydrogels are that the gelling process is achieved under physiological conditions and the encapsulation of drugs, including proteins and even live mammalian cells, are easily accomplished [14]. In the alternative hybrid approach, networks are generated from synthetic polymers with oligonucleotides as cross-linking units. Such materials are sensitive to temperature [15] and DNA host molecules [16] and change their mechanical properties reversibly upon the addition of DNA sequences [17]. The latter trigger can even be employed to release incorporated semiconductor quantum dots on demand [18]. More-regular structures with high microscopic order are obtained by cross-linking gold nanoparticles with oligonucleotides into crystalline states. By choosing the sequence it is even possible to generate different crystal structures [19].

In this contribution we present a new method for the generation of unprecedented ***all-organic*** conjugated-polymer nanoparticle networks guided by DNA, which are based on a hierarchical self-assembly process. First, microphase separation of amphiphilic block copolymers induced the formation of spherical nanoobjects. As a second ordering principle, DNA base pairing was employed for controlled spatial definition of the conjugated-polymer particles within the bulk material. These networks offer the flexibility and the diversity of soft polymeric materials. As such, simple chemical methodologies could be applied in order to tune the network's electrical, optical and mechanical properties.

In recent years, several methods have been developed to generate linear DNA block-copolymer (DBC) structures by "grafting–onto" strategies connecting the termini of the biological and the organic polymer segments [20-22]. For water-soluble polymers, efficient attachment to the nucleic-acid component was achieved in water or buffer solutions, while hydrophobic polymers were coupled in high yields on the solid phase.

## Results and discussion

Since our aim was to produce electronically-active, nanostructured bulk materials with the help of DNA, we used a DNA block copolymer with the hydrophobic segment, a polyfluorene derivative covalently linked to single stranded DNA (22mer). DNA-*b*-PF micelles were generated simply through dissolving the polymer [23] in a buffer solution followed by heating to 95°C and cooling to room temperature overnight. The resulting spherical aggregates with cores consisting of polyfluorene and shells composed of single-stranded (ss) DNA were visualized on a mica surface by atomic-force microscopy (AFM), showed an average height of 8 ± 3 nm (see Additional file 1).

**Figure 1:**
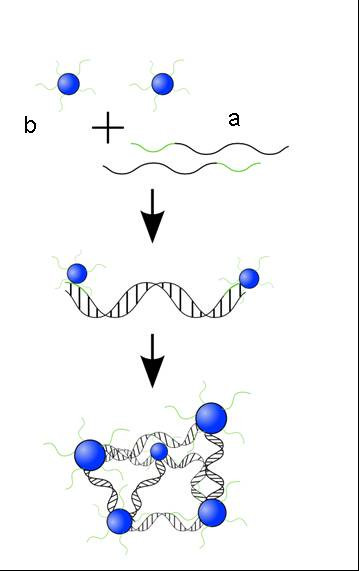
First design of higher-ordered structures: A rigid duplex, consisting of 120 bp and two terminal 24mer overhangs (a) which were complementary to the DNA present in the corona of DBC micelles (b).

**Figure 2:**
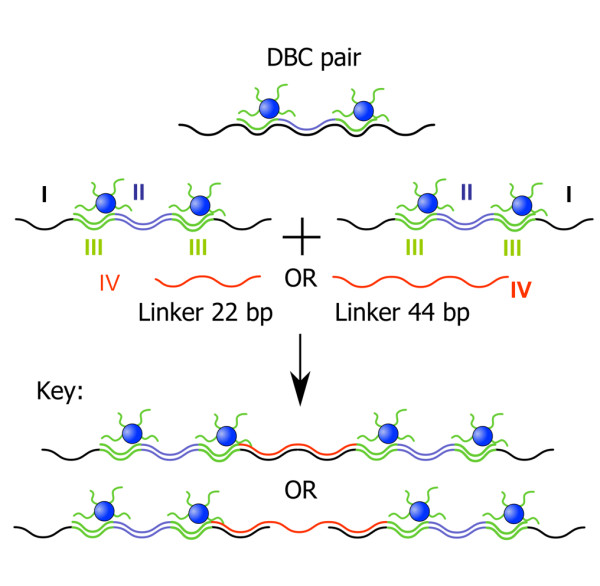
**AFM and HRTEM images of DNA-conjugated polymer nanoparticle network as described in scheme 1.** Insets show high magnification of the scanned area. a-c) AFM scans of the linear (scale bar 200 nm), 2-D (scale bar 500 nm) and 3-D materials (scale bar- 1.9 μm, inset 400 nm), respectively and d-f) the corresponding HRTEM pictures (scale bars: d,e. 1 μm, 100 nm, f. 1 μm and 50 nm).

On the basis of this self-assembled building block, higher-ordered structures were fabricated by hybridization (Figure 1). A rigid duplex was designed consisting of 120 base pairs (bp) and two terminal 24mer overhangs that were complementary to the DNA present in the corona of DBC micelles . After mixing micelles and linker strands the resulting structures were analyzed by AFM and Transmission Electron Microscopy (TEM). As revealed by the former technique, linear assemblies, two-dimensional sheets and three-dimensional fractals were formed and are shown in Figures 2a, 2b and 2c, respectively.

For the one- and two-dimensional structures, either in tapping mode or in phase mode, micelle cores were identified as bright spots. Moreover, both types of structures showed characteristic spacing of the microphase-separated conjugated polymers within the DNA matrix. The duplex of 166 bp bridging the core of the micelles exhibits a length of 56.4 nm on the assumption of 0.34 nm per nucleotide. Statistical analysis of the characteristic spacing of conjugated polymer cores gave distances of 50 ± 7 and 66 ± 7 nm for one- and two-dimensional assemblies, respectively, which is in good agreement with the theoretical value (see Additional 1 Figure S 1 and Fig. S 2). For the 3-D fractals, microdomains within the material could be measured only at the edges of the sample, exhibiting average distances of 80 ± 5 nm (Figure 2c, inset).

While AFM gives information about the ordering on the surface of the material, TEM allowed investigation of the bulk structure. For the TEM study, DNA complementary to that of the corona of the DBC micelles was immobilized on a gold-coated grid *via* an Au-S-bond, with the use of a previously published procedure [24,25]. Interestingly, high contrast was observed between micelle cores and DNA within the sample. This might be attributed to the semiconducting properties of polyfluorene of the DBCs and is in sharp contrast to other block-copolymer systems investigated by TEM [26]. As for AFM, one-dimensional structures could be well visualized by TEM. The micelle cores that were separated through linker DNA, appeared as dark spots (Figure 2d). On average, the PFO domains were 800 nm apart,, indicating the formation of aggregates.

Following this, the two-dimensional structures were investigated by TEM at lower and higher magnifications. The low-magnification images of the DNA micelle networks show films of varying thickness appearing as shades of grey (Figure 2e). While these images did not allow the identification of individual DBC particles, such structures were visible at higher magnification (Figure 2f). The micelle cores appeared as dark spots scattered within a matrix of brighter contrast. Different film thicknesses correspond to areas with different grey scale. It should be noted that quantitative distance analysis cannot be performed for imperfect crystals as exist in our sample. However, TEM undoubtedly proves microstructure formation of DBC micelles induced by linker DNA (Figure 2e, inset).

The 3-D fractals appeared as darker areas in the TEM picture at lower magnification (Figure 2f). At higher magnification, individual particles could be identified at the edges of the structures (Figure 2f inset), confirming the observation of the AFM measurements. The presence of 1-D chains, 2-D sheets and 3-D fractals have been reported for inorganic nanoparticles equipped with various covalently attached surface functionalities including DNA [27]. Our structural analysis reveals similar behavior; however, the materials presented here consist exclusively of organic building blocks and, even more important, rely on two self-assembly processes, namely, Watson-Crick base-pairing of DNA and microphase separation of the organic polymer segment in an aqueous environment.

**Figure 3:**
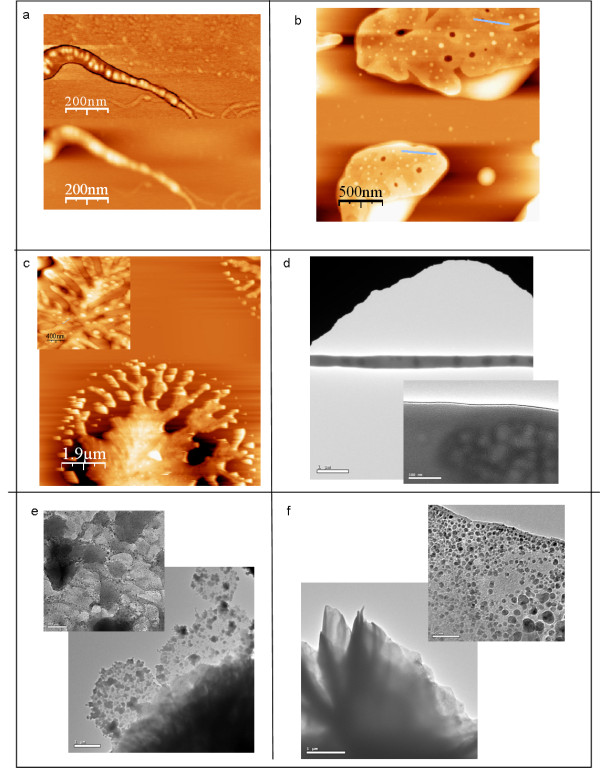
**Second design of higher-ordered structures: Pairs of micelles III are assembled with template I and sequence II.** Subsequent hybridization with connector oligonucleotides IV leads to superstructures (see text for details).

**Figure 4:**
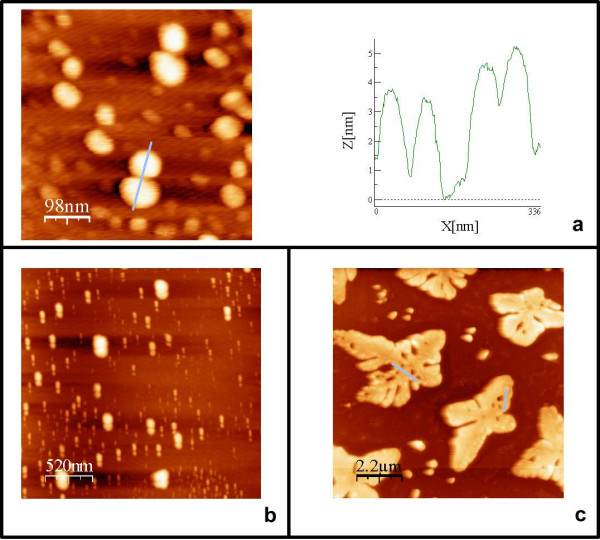
**AFM analysis of the second type of DBC micelle superstructure according to scheme 2.** Each image represents DBC architectures with different linker lengths, a) no linker, b) 24 bp- and c) 44 bp linker IV.

To demonstrate the flexibility of our approach, we fabricated a second type of DNA hybrid material with different structural features. The system contained four building blocks (Figure 3). The sequence of the 115mer template I was chosen in such a way that hybridization with sequence II results in a central double-stranded DNA part, which is flanked by two annealing sites for the DBC micelles III (2x22 bp). The remaining 44mer overhangs of the template allow formation of extended structures by hybridization with sequence IV, a 24mer. After annealing of all components in a single hybridization reaction, the resulting structures were analyzed by AFM on a mica surface. The AFM images suggest the formation of one-dimensional extended DNA chains decorated with pairs of DBC micelles (Figure 4a and 4b).

Statistical analysis of the separation of particles revealed two characteristic distances. The first was 7 ± 2 nm, which can be ascribed to the spacing of two DBC micelles by sequence II. This separation is in good agreement with the calculated value of 7.7 nm. The second characteristic distance was found to be 13 ± 2 nm. This can be explained by linking DBC micelle pairs by sequence IV. Again good agreement with the calculated value (14 nm) was observed.

Finally, we varied the size of sequence IV, which connects template-strand I. When the length of IV was increased from 24 to 44 bp, completely different structures were detected by AFM, namely, dendritic architectures. Such surface topologies have been found for other polymers as well [28]. This great structural difference can be attributed to the changed connection between template-strands I. When sequence IV is composed of 22 bp, exclusively ds DNA is present, whereas when IV consists of 44 bp, ds stretches alternate with ss stretches of 22 bp. We ascribe the different structures to the difference in persistence length of the ds DNA (50 nm) versus that of ss DNA (2-3 nm). In similar experiments with polymers, the rigidity of the backbone was found to be an important parameter determining the structural features of large assemblies. In a separate study, we will report on the detailed growth mechanism of the dendritic structures.

## Conclusions

In summary, we have shown the fabrication of 1-, 2- and 3-D structures composed of DNA and DBCs. Common to all morphologies is the integrity of the micelles consisting of DBCs. Moreover, the micelles represent not only structural features but act as cross-linking units as well. Most noteworthy is the structural diversity of the morphologies controlled by variation of the DNA sequences. One cannot expect to realize the same degree of order as that obtained for pristine DNA assemblies based on the tile and origami approaches [29]. The same holds true for networks generated by oligonucleotide functionalized gold nanoparticle networks. There is a striking difference between the bioorganic structures presented here and the all DNA and bioinorganic assemblies. The DNA conjugated-polymer superstructures rely on two self-assembly processes, while in pristine DNA and the Au nanoparticle-DNA assemblies only Watson-Crick base pairing is responsible for superstructure formation. In this respect, the materials presented here should be compared to block-copolymers which have also been reported containing microphase-separated semiconducting domains [30,31]. We have demonstrated that typical morphologies of conventional block copolymer systems can be extended by the utilization of DNA block copolymers allowing additional structure control by the DNA segment.

## Methods

### DBC micelles

poly(9,9-dioctylfluorene) was functionalized with one terminal hydroxyl group as described previously^[23]^. After conversion of 1 to the phosphoramidite polymer (2), the desired diblock-copolymer architecture, DNA-*b*-PF (3) was obtained by grafting 2 onto the immobilized DNA and incubating with concentrated ammonia to achieve liberation from the solid support and de-protection of the nucleobases. The nucleic acid segment was composed of the 22mer sequence 5’-CCTCGCTCTGCTAATCCTGTTA-3’ with a molecular weight of 6670 g/mol while the organic polymer consisted of polyfluorene with a total number average molecular weight of 5000 g/mol.

### Pairs

0.1 nmol of sequence 1 was immersed in 500*μL* PBS buffer solution (pH = 7.4) and added to 0.2 nmol F8PB1147 block copolymer micelles solution immersed in 100*μL* PBS buffer and left for 24 h at room temperature for hybridization. After hybridization took place, 50*μL* of the medium were deposited on a mica surface, left for 4 h and dried with nitrogen gas.

### 3D network

For the preparation of the double-strand DNA connector, 71.4*μL*of a solution containing 0.1 nmol of sequence 3 was mixed with 38.46*μL* of 0.1 nmol of sequence 4. The block-copolymer solution (0.1 nmol in 10*μL*) was diluted to a volume of 0.5 mL with PBS buffer. The two solutions were then mixed and deposited on a mica surface.

### HR-TEM imaging

TEM measurements were performed on gold-coated TEM grids that were immersed in 100*μL* of a solution of the network of interest of complementary thiolated ssDNA strands (0.5 nmol in 100*μL*). These grids were kept in solution for 24 h and then dried with nitrogen gas before imaging.

#### DNA sequences

##### Sequence 1

5' AATCATACGTACTCAACTGCTG GGAGCGAGACGATTAGGACAAT AACTTGGGTATGCTGTCAGATGGCTCG GGAGCGAGACGATTAGGACAAT AATCATACGTACTCAACTGCTG 3'

##### Sequence 2

5' CGA GCC ATC TGA CAG CAT ACC CAA GTT3'

##### Sequence 3

5'TGG CCA CGA ACA AAC AAG ACA AGA GAG TAA GTC TGA TCT GGA GAG GTC GGA AAT CAT AGA AAC CAC ACG AAT GAT AAG GCA TGG AGG TAA AAG GCA TCA ATA ACA GGA TTA GCA GAG CGA GG 3'

##### Sequence 4

5' TTG ATG CGT TTT ACC TCC ATG CCT TAT CAT TCG TGT GGT TTC TAT GAT TCT CGA CCT CTC CAG ATC AGA CTT ACT CTC TTG TCT TGT TTG TTC GTG GCC ATA ACA GGA TTA GCA GAG CGA GG3'

##### Thiolated DNA

5' (Thiol C6) ATA CCC ACG CCG AAA CAA GC

## Abbreviations

DBC, DNA block copolymer; TEM, Transmission Electron Microscopy.

## Competing interests

The authors declare no competing interests.

## Authors’ contributions

ED and KL carried out the AFM studies and the DNA hybridization. DP synthesized the micelles. All participated in the design of the experiments, coordination and helped to draft the manuscript. All authors read and approved the final manuscript.”

## Supplementary Material

Additional file 1 Atomic-Force-Microscopy analysis and statistical data of the spacing lengths of the network are presented. Click here for file
