# Supplementary material for: DNA-nanoparticle assemblies go organic: Macroscopic polymeric materials with nanosized features
Source: J Nanobiotechnology. 2012 May 30;10:21. doi: 10.1186/1477-3155-10-21 (PMC3408339; doi:10.1186/1477-3155-10-21)
Supplement: Additional file 1 — Atomic-Force-Microscopy analysis and statistical data of the spacing lengths of the network are presented. [file 1477-3155-10-21-S1.doc]

# *Supporting Information*

DNA-Nanoparticle Assemblies Go Organic: Macroscopic Polymeric Materials with Nanosized Features

Elad D. Mentovich^#^, Konstantin Livanov^#^, Deepak K. Prusty^§^, Mukules Sowwan^$*^, Shachar Richter^#^*

*Nanotechnology Research Laboratory, Materials Engineering Department, Al-Quds University, East Jerusalem, Palestinian Authority.*

*University of Groningen, Zernike Institute for Advanced Materials, Nijenborgh 4, 9747 AG Groningen, The Netherlands.*

*School of Chemistry, Raymond and Beverly Sackler Faculty of Exact Sciences and Research Institute for Nanoscience and Nanotechnology, Tel-Aviv University, Ramat Aviv, Tel-Aviv 69978, Israel*

E-mail: [srichter@post.tau.ac.il](mailto:srichter@post.tau.ac.il)

1. Atomic-force microscopy analysis of amphiphilic DNA-*b*-PF.

Figure S1 show typical AFM scan line and spacing of DNA-b-PF micelles on a mica surface.

Figure S1. AFM image of DNA-*b*-PF.

2. Statistical analysis of the characteristic spacing

Figure S2, S3 show typical AFM scan line and spacing of 2D and 1D structures.

Figure S2. AFM analysis of the 2D network.

Figure S3. AFM analysis of a 1D network.
